# Early Visual Detection of Wheat Stripe Rust Using Visible/Near-Infrared Hyperspectral Imaging

**DOI:** 10.3390/s19040952

**Published:** 2019-02-23

**Authors:** Zhifeng Yao, Yu Lei, Dongjian He

**Affiliations:** 1 College of Mechanical and Electronic Engineering, Northwest A&F University, Xianyang 712100, China; yzf8466@163.com (Z.Y.); ly759059219@163.com (Y.L.); 2 Key Laboratory of Agricultural Internet of Things, Ministry of Agriculture and Rural Affairs, Xianyang 712100, China; 3 Shaanxi Key Laboratory of Agricultural Information Perception and Intelligent Service, Xianyang 712100, China

**Keywords:** wheat stripe rust, hyperspectral imaging, incubation period, SPAD, spatial distribution

## Abstract

Wheat stripe rust is one of the most important and devastating diseases in wheat production. In order to detect wheat stripe rust at an early stage, a visual detection method based on hyperspectral imaging is proposed in this paper. Hyperspectral images of wheat leaves infected by stripe rust for 15 consecutive days were collected, and their corresponding chlorophyll content (SPAD value) were measured using a handheld SPAD-502 chlorophyll meter. The spectral reflectance of the samples were then extracted from the hyperspectral images, using image segmentation based on a leaf mask. The effective wavebands were selected by the loadings of principal component analysis (PCA-loadings) and the successive projections algorithm (SPA). Next, the regression model of the SPAD values in wheat leaves was established, based on the back propagation neural network (BPNN), using the full spectra and the selected effective wavelengths as inputs, respectively. The results showed that the PCA-loadings–BPNN model had the best performance, which modeling accuracy (*R_C_*^2^) and validation accuracy (*R_P_*^2^) were 0.921 and 0.918, respectively, and the RPD was 3.363. The number of effective wavelengths extracted by this model accounted for only 3.12% of the total number of wavelengths, thus simplifying the models and improving the rate of operation greatly. Finally, the optimal models were used to estimate the SPAD of each pixel within the wheat leaf images, to generate spatial distribution maps of chlorophyll content. The visualized distribution map showed that wheat leaves infected by stripe rust could be identified six days after inoculation, and at least three days before the appearance of visible symptoms, which provides a new method for the early detection of wheat stripe rust.

## 1. Introduction

Wheat stripe rust, caused by *Puccinia striiformis f. sp. tritici* (*Pst*), and characterized by strong outbreaks, high incidence, wide occurrence area, and large economic losses, is one of the most prevalent wheat diseases, responsible for severe yield decreases worldwide [1,2,3]. China is the world’s largest stripe rust epidemic area, and wheat yields can be reduced by 10–20% during wheat stripe rust epidemic years, and by more than 50%, or even 100%, in exceptionally epidemic years [4,5,6]. The infection process of *Pst* can be divided into the phases of contact, invasion, incubation, and incidence [7,8]. In the incubation period, uredospores lurk in the interior of wheat leaves and absorb nutrients from the host to rapidly grow and reproduce; however, symptoms do not show, making it impossible for people to perceive the infection. Once the conditions are appropriate, a large area of stripe rust will break out and become prevalent. If the effective diagnosis of wheat stripe rust leaves can be carried out accurately and quickly during the incubation period, prevention and control measures can be taken as early as possible, and the losses caused by the disease will be greatly reduced. Additionally, the early detection of disease can also reduce the application of pesticides and lower environmental pollution [9]. Therefore, it is of great importance to study the early detection of wheat stripe rust during the incubation period.

To monitor and detect wheat stripe rust, scouting methods by visual inspection of the foliage in the field are traditionally employed. However, this method is not only time consuming and labor intensive, but also somewhat subjective [10]. Other types of methods for plant diseases diagnosis and detection include the microscopic evaluation of morphology features to identify pathogens [11], as well as molecular diagnostic techniques [12], but these methods demand experienced individuals with well-developed skills in disease detection and are thus subject to human bias. Consequently, a convenient and accurate method is urgently needed for the detection of plant diseases.

Hyperspectral imaging is a new crop monitoring technique that has been developed in recent years, which combines conventional imaging and spectroscopy to acquire both spatial and spectral information from the detected target at the same time [13]. As a result of the combined features, it has been widely used in crop nutrient detection [14,15,16], diseases diagnosis [17,18,19], and growth status monitoring [20,21,22], amongst others. In terms of wheat disease, many studies on leaf and canopy scale monitoring have been conducted using ground and aerial hyperspectral remote sensing technologies. For example, Huang et al. [23] adopted in-situ spectral reflectance measurements and airborne hyperspectral imaging to quantify the disease index of yellow rust in wheat on a canopy scale. Zhang et al. [24] used in-situ hyperspectral data for detecting and discriminating stripe rust disease from nutrient stresses. Lei et al. [25] carried out hyperspectral measurements of the severity of stripe rust on individual wheat leaves at the grain filling stage. Liang et al. [26] used hyperspectral imaging technology to quantitatively identify wheat stripe rust and powdery mildew with a classification accuracy of more than 92%. These studies described above point clearly as to the potential of hyperspectral imaging for quantifying the incidence or severity of stripe rust in wheat. However, these were realized after the diseases could be seen by the naked eye, whereas the diagnosis and detection of wheat stripe rust under early stress without symptoms has rarely been reported.

In the early stages of stripe rust infection, pathogenic fungi absorb the necessary nutrients and water from the host for reproduction and expansion, resulting in significant changes in the physiological conditions of the wheat, such as cell structure and chlorophyll content [27], thus affecting the spectral reflectance of wheat leaves. Therefore, chlorophyll content can be used as an important indicator to monitor plant growth status and disease stress [28,29,30,31]. More recently, researchers have investigated the link between chlorophyll content or chlorophyll-related reflectance wavebands and crop diseases. Gitelson et al. [32] proved that reflectance at 700 nm (red wavelength) and 550 nm (green wavelength) was highly correlated with total leaf chlorophyll content. Qing et al. [33] found that the infection of stripe rust led to the decrease of chlorophyll content and photosynthetic capacity in wheat leaves, thus affecting the growth and yield of wheat. He et al. [34] further concluded that there was a highly significant negative correlation (*r* = −0.88) between the stripe rust disease index and chlorophyll content of leaves, so the change in chlorophyll content could be used as an indicator to determine whether winter wheat was subjected to stress. Although the above-mentioned studies achieved some useful results, it is still uncommon to detect stripe rust through the fusion of spectral information and chlorophyll content of the wheat leaves, especially in the early stages of disease stress. 

In this study, the hyperspectral images of wheat leaves infected by stripe rust for 15 consecutive days were collected, and the chlorophyll content were measured simultaneously in the laboratory. The objective of this study was to investigate the possibility of the rapid and accurate detection of stripe rust by combining spectral information with the chlorophyll content of wheat leaves, especially in realizing the early monitoring of wheat disease before the occurrence of visible susceptibility characteristics with the naked eye. The results may provide theoretical guidance for the early detection of crop disease.

## 2. Materials and Methods

### 2.1. Sample Preparation

Mingxian 169 wheat, which is highly susceptible to *Pst*, was grown in plastic pots (7 × 7 × 8 cm) at a density of 10–15 seeds in each pot, in a rust-free growth chamber (16 ± 3 °C, 16 h light/8 h darkness) in the State Key Laboratory of Crop Stress Biology for Arid Areas and College of Plant Protection, Northwest A&F University, China. When the seedlings had grown two or three leaves, the first wheat seedling leaf was gently rubbed with a clean moistened finger to remove the waxy layer from the leaf surface. *Pst* pathogens naturally infected leaves (from living tissues) were collected and evenly smeared on the first wheat seedling leaf with a brush, and the inoculation zones were labeled with a marker. There was a total of 40 wheat seedling pots, of which 35 pots were inoculated with the above method, and the remaining five pots without inoculation were used as healthy controls. The inoculation group and healthy control group were placed in separate plastic boxes and covered with wet polyethylene bags to maintain 100% relative humidity, and were stored for 24 h at 10 °C in a dark chamber. Immediately after incubation, plants were transferred to a clean growth chamber set to a diurnal cycle of 16 °C for the 16 h light period, and 13 °C for the 8 h dark period [35]. Ten days later, the inoculated wheat plants began to show scabs successively, indicating that the inoculation of stripe rust was successful. From day 1 to day 15 after inoculation, 15 leaves were randomly selected from the inoculated wheat seedlings at 15:00 every day for hyperspectral image collection and chlorophyll content measurement. Additionally, 15 leaves were randomly selected from the wheat seedlings that had not been inoculated as the healthy control.

### 2.2. Hyperspectral Image Acquisition and Calibration

**Figure 1:**
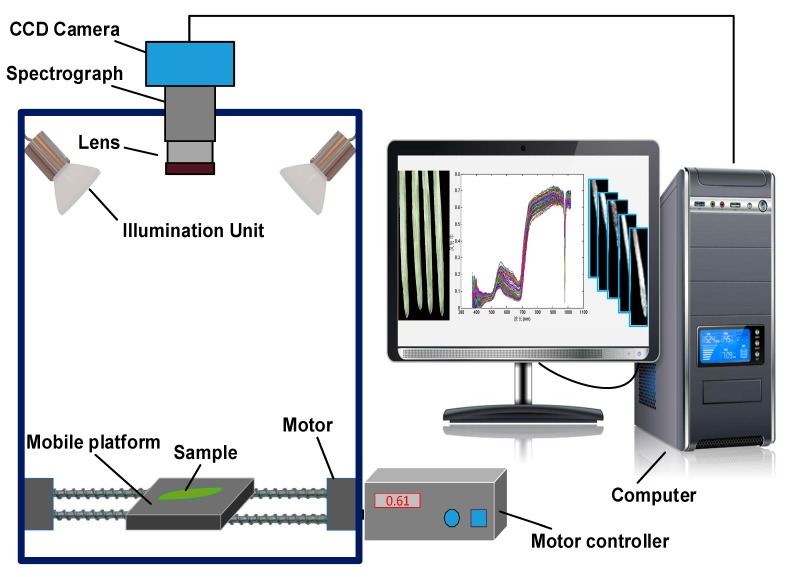
The schematic diagram of the hyperspectral imaging system.

A line-scanning hyperspectral imaging system, as shown in Figure 1, in the spectral range of 375–1017 nm was used to acquire the hyperspectral images of wheat leaves. The system mainly consisted of a visible/infrared imaging spectrograph (ImSpector V10E, Spectral Imaging Ltd., Oulu, Finland), a high-performance 8-bit charged couple device (CCD) camera (OPCA05G, Hamamastu, Shizuoka Prefecture, Japan) coupled with a zoom lens (OLES23, Specim, Spectral Imaging Ltd. Oulu, Finland), an illumination unit equipped with four 100 W halogen lamps at angle of 45° (HSIA-LSTAIF, Zolix Instruments Co., Ltd., Beijing, China), a mobile platform (PSA200-11-X, Zolix Instruments Co., Ltd. Beijing, China), a camera obscurer, a stepper motor, and a computer with data acquisition software Spectra SENS (Spectral Imaging Ltd. Oulu, Finland). The hyperspectral imaging system was set up in the laboratory, with the room temperature of 28 ± 1 °C and relative humidity of 50%. Wheat leaves were placed on the mobile platform operated by the stepper motor at a speed of 14 mm/s. To obtain clear and undistorted hyperspectral images, the distance between sample and lens was 65 cm, and the exposure time was 5 ms during the image acquisition. Each image was acquired as a three-dimensional image (*x*, *y*, *λ*), which included 320 × 250 pixels in spatial dimension (*x*, *y*) and 256 spectral bands from 375 to 1017 nm.

Due to the existence of dark current in the CCD camera and the uneven intensity of illumination in different bands, some bands with a weaker light intensity contained bigger noises. Hence, the raw hyperspectral images (*I*_raw_) required to be calibrated and the calibration process was performed according to the following equation:

(1) $$I_{\mathrm{calibrated}}=\frac{I_{\mathrm{raw}}-I_{\mathrm{dark}}}{I_{\mathrm{white}}-I_{\mathrm{dark}}}$$

where *I*_calibrated_ was the calibrated image, *I*_raw_ was the raw hyperspectral image, *I*_white_ was the white reference image with 99% reflectance acquired from a white reference ceramic tile, and *I*_dark_ was the dark reference image with 0% reflectance, which was obtained with the camera lens completely covered with an opaque cap.

Next, the calibrated images were used as the basis for subsequent analysis, including the extraction of spectral information, the selection of relevant wavelengths, and the establishment of regression models.

### 2.3. Measurement of SPAD Values of Leaves

The chlorophyll content of the wheat leaves were measured by a handheld SPAD-502 chlorophyll meter (Minolta Camera Co., Osaka, Japan). The SPAD-502 chlorophyll meter, a simple and portable diagnostic tool, can measure the greenness or relative chlorophyll content by acquiring the absorbance of leaves in the red and near infrared regions at individual points in a non-destructive way [36]. The SPAD approach uses point-based measurements, and can only provide SPAD values in a small area. Therefore, eight SPAD readings were obtained from each wheat leaf, and the average value was taken as the SPAD value of the tested sample. Since the clamping of the chlorophyll meter damaged some leaves, after removing the injured samples, the number of samples with each latent day ranged from 10 to 15. A total of 184 SPAD values for 15 consecutive days were obtained, one-to-one corresponding to the hyperspectral data.

### 2.4. Extraction and Preconditioning of Spectral Data

**Figure 2:**
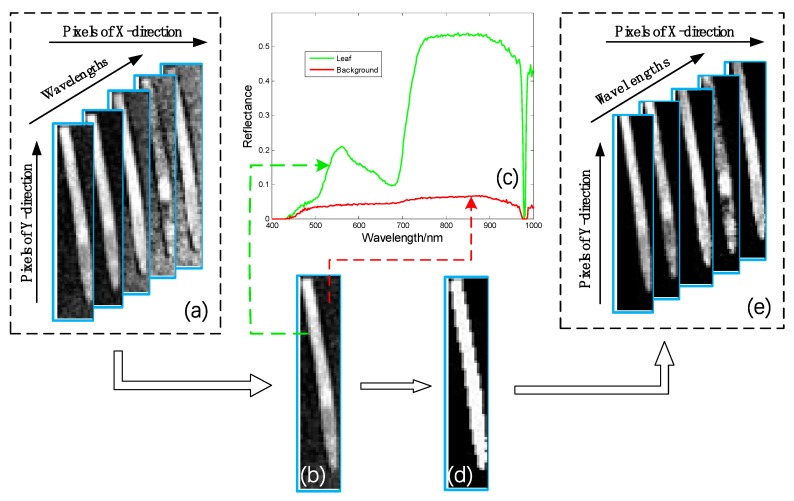
Procedure for image segmentation.

In order to accurately separate the leaf and background regions in the hyperspectral images of wheat leaves to reduce the background error, ENVI + IDL 5.1 software (ITT Visual Information Solutions, Boulder, CO, USA) was adopted for image segmentation, as shown in Figure 2. Since the reflectivity of the background was relatively stable in the range of 500–600 nm and had a great difference with the spectral reflection curve of the leaf region, the segmentation threshold was set between the background reflectivity and the leaf reflectivity, which could divide the two parts. Through comparative analysis, it was found that the spectral reflectance of the background and the blade was significantly different at 560 nm (Figure 2c), so the segmentation threshold was set as 0.12, which was greater than the maximum reflectance of the background and less than the minimum reflectance of the leaf. The leaves and background were successfully segmented, and the mask of the leaves were obtained, as shown in Figure 2d. Meanwhile, logic and calculations were conducted to mask every hyperspectral image with a leaf mask, and target regions of the leaf hyperspectral images were identified (Figure 2e). The leaf regions of the 184 samples used in this paper were all extracted according to the above method, and taken as regions of interest (ROIs), and the average spectra of each pixel in ROI was taken as the spectral reflectance of the corresponding sample. According to this procedure, 184 spectral reflectance values were obtained and imported into the MATLAB 2016a software (MathWorks, Natick, MA, USA) for further data analysis.

Preconditioning of the hyperspectral data was required to extract the maximum effective information from the hyperspectral data and to eliminate the influence of adverse factors such as noise, spectral line translation, and stray light. The common processing methods of Savitzky–Golay (SG) smoothing [37], multiple scatter correction [38], standard normal variate transformation [39], and first and second derivative [40] were tested. SG smoothing was found to eliminate the spectral noise while maintaining the spectral characteristics; therefore, SG smoothing was selected for the data treatment throughout the study.

Considering that the partition of the calibration set and the prediction set has a great influence on the model accuracy, this paper selected the *X*–*Y* distances sample set partitioning method (SPXY) [41] to divide the samples by a ratio of roughly 3:1, and the samples were divided into 140 calibration sets and 44 prediction sets.

### 2.5. Selection of Effective Wavebands

Full spectra contains all of the spectral information with large data files, information redundancy, and multiple collinear variables. Therefore, the full spectra would add to the complexity of the model, reduce the computation speed, and affect the model accuracy. Therefore, it is necessary to find the effective wavelengths (EWs) that play a key role in modeling.

Principal component analysis (PCA) [42] is an unsupervised technique and has a wide application in reducing the dimension of multivariate datasets. Meanwhile, the score plot of principal components (PCs) is used to reveal the features of the variable distribution, and the loadings plot of PCs can exhibit the importance of different variables [43]. Generally, the loadings of the first few PCs are used for optimal wavelength selection.

The successive projections algorithm (SPA) [44] is a forward variable selection method that employs simple projection operations on wavelengths to select subsets of variables with minimum redundancy and collinearity. In this way, it can effectively eliminate information overlap among the wavebands, minimize the variables, and improve the speed and efficiency of the model. 

The selection of effective wavebands carrying the most valuable and authentic information is a challenging task in hyperspectral data analysis. In this study, the above two methods were used and compared for effective wavebands selection.

### 2.6. Modeling Approach

Back propagation neural network (BPNN) is a canonical feed-forward network where an error signal was fed back through the network. Weights among the simple processing units of nodes were adjusted by iterating input patterns throughout the network until the error between the network output and the targeted output was minimized [45]. The three-layer perceptron BPNN can discriminate any arbitrarily complex region, and can approximate any given function with arbitrary precision [46]. Therefore, this paper used a three-layer perceptron BPNN to establish a calibration model for chlorophyll content evaluation. The BPNN consisted of three layers: an input layer, an output layer, and a hidden layer. The number of input nodes was equal to the number of input variables. The node of hidden layer was set to 8 through multiple comparative experiments. The number of output nodes was equal to 1, corresponding to the chlorophyll content. Tangent sigmoid and linear function were used as the transfer function. Meanwhile, over-fitting was avoided by using a Bayesian regularization training algorithm. The network was trained for at least 20,000 epochs.

### 2.7. Evaluation of Models

The model performance was evaluated using the determination coefficient (*R_P_*^2^) of the prediction set, the root mean square error of the prediction set (RMSEP), the determination coefficient of calibration set (*R_C_*^2^), root mean square error of the calibration set (RMSEC), and the residual predictive deviation (RPD) [47]. A good model should have high values of *R_P_*^2^ and *R_C_*^2^, and low values of RMSEC and RMSEP. RPD represents the prediction ability of the model; values above 2.0 mean that the model has a good prediction accuracy. Moreover, the number of input variables in the model was used to evaluate whether the model was simple or not; fewer input variables in the model suggested a simpler model. Each model run 10 times, and the means of the performance indices were taken as the experimental results.

### 2.8. Visualization of Distribution Maps

It is quite difficult to measure the chlorophyll content for every part of the sample using chemical analysis methods or a chlorophyll meter, and the advantage of hyperspectral imaging makes it feasible to predict the chlorophyll content of each pixel of the sample within hyperspectral images by the developed calibration models. The spatial distribution of the chlorophyll content could be generated with the established quantitative model combined with image processing. In particular, the spatial position of each pixel along with its chlorophyll content was used to form the spatial distribution maps in this study.

## 3. Results and Discussion

### 3.1. Spectral Features of Wheat Leaves at Different Days Post-Inoculation

**Figure 3:**
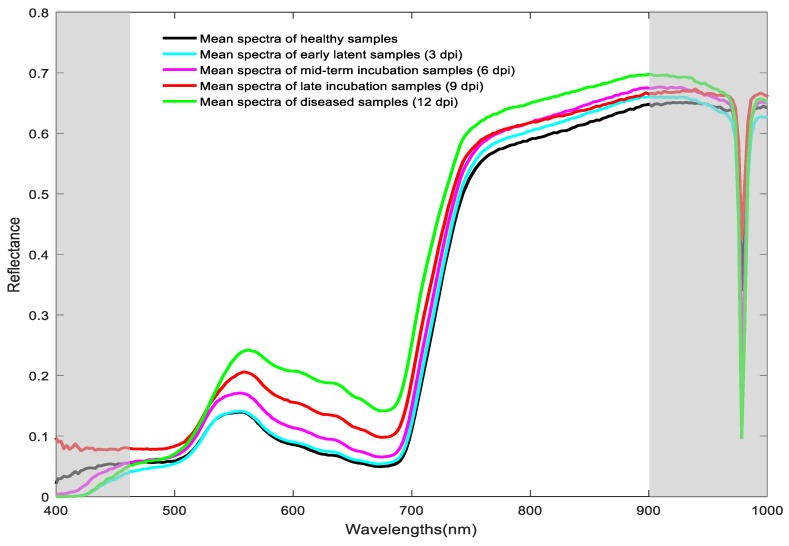
Mean spectral reflectance curves of wheat leaves with different days post-inoculation (dpi).

Figure 3 shows the spectral reflectance curve of healthy wheat leaves and wheat leaves infected by stripe rust at different days post-inoculation. The spectral curve conforms to the spectral characteristics of green plants [48,49], namely, the wave peak near 550 nm is associated with the strong reflection of chlorophyll, and the wave trough near 680 nm is due to the strong absorption of chlorophyll. The spectral reflectance rose sharply at 680–750 nm, generally referring to the “red edge” [50]. There are some differences in the reflectance spectra of wheat leaves with a different number of days post-inoculation. The reflectance of wheat leaves infected with stripe rust was higher than that of the healthy leaves, and the reflectance gradually increased with the increase of days post-inoculation, which was largely consistent with the result trend observed by Wang et al. [51]. Specifically, the distinct valley at 970 nm was an artifact of the spectral data acquisition process. In order to excluded this artefact and reduce the heavy noises at both ends of the spectral range, spectrum data below 450 nm and above 900 nm were discarded, leading to spectra within the range of 450–900 nm with 180 bands for further analysis.

### 3.2. SPAD Analysis of Wheat Leaves at Different Days Post-Inoculation

**Figure 4:**
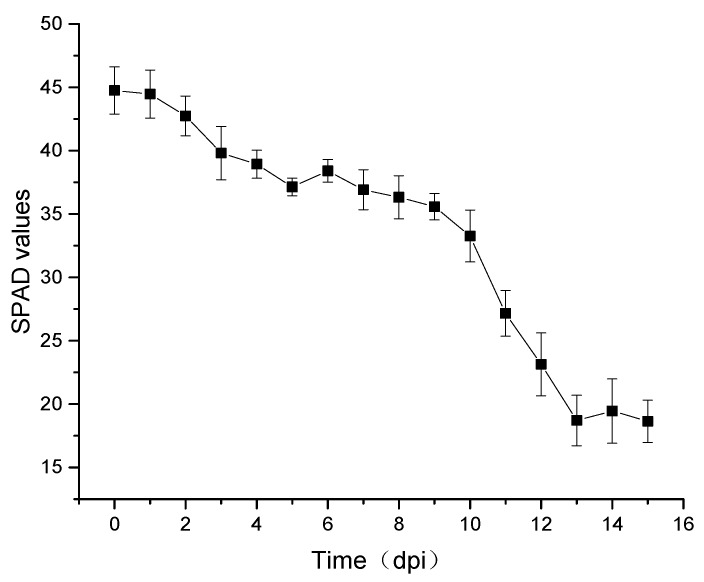
The average SPAD curve of wheat leaves during different days post-inoculation.

Figure 4 shows the SPAD average curve and standard deviation of wheat leaves at different days post-inoculation, where the abscissa is the number of days post-inoculation and the ordinate is the SPAD values. 

The SPAD value gradually decreased in the first nine days post-inoculation (dpi) of stripe rust. From day 10 to 13 after inoculation, the SPAD value decreased sharply, ranging from 33.71 to 18.76. This period represented the stage when symptoms first appeared in leaves; the leaves were obviously chlorotic, the tissue cells were destroyed, and the spores of stripe rust spread rapidly on the leaf surface. After 14 dpi, the leaves atrophied, curled, or even died, and the SPAD value changed very little.

### 3.3. Selection of Effective Wavelengths

**Figure 5:**
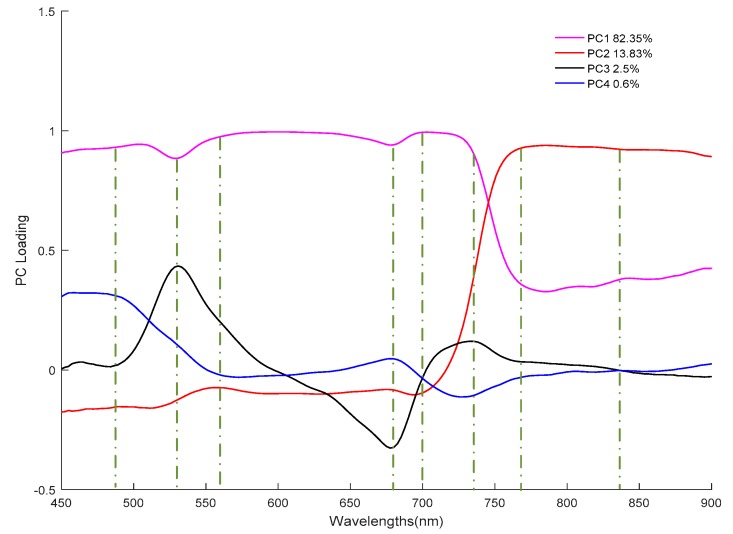
Effective variables selected by principal component analysis (PCA).

In this study, PCA was employed to find multiple appropriate wavelengths at 450–900 nm that could simplify the model structure and improve model accuracy. From the calculation, the first four principal components (PCs), accounting for 82.35%, 13.83%, 2.5% and 0.6% of the total variance, respectively, were selected for further analysis, because more than 99.28% of variation was retained in first four PCs. Since each PC score is a linear combination by each spectral point multiplied by the corresponding loading, the wavelengths located at local maximal values (peaks) or minimal values (valleys) of the loadings curve have a more important contribution to the PC score [52]. Therefore, the loadings curves of the first four PCs, as shown in Figure 5, were subjected to selecting the effective wavebands. Loadings at local peaks and valleys can be regarded as characteristic loadings and the corresponding wavelengths were considered as key wavelengths for further regression models. Thus, eight effective wavelengths of 484, 530, 560, 680, 700, 738, 766, and 837 nm were preferably selected and indicated by a broken line. The number of selected dielectric variables was 3.12% of the 256 variables in the full spectra.

**Figure 6:**
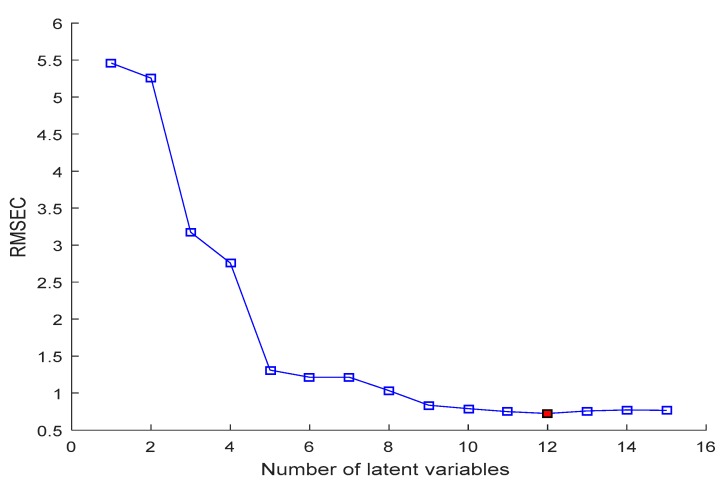
Changed RMSEC with increasing number of variables in SPA.

**Figure 7:**
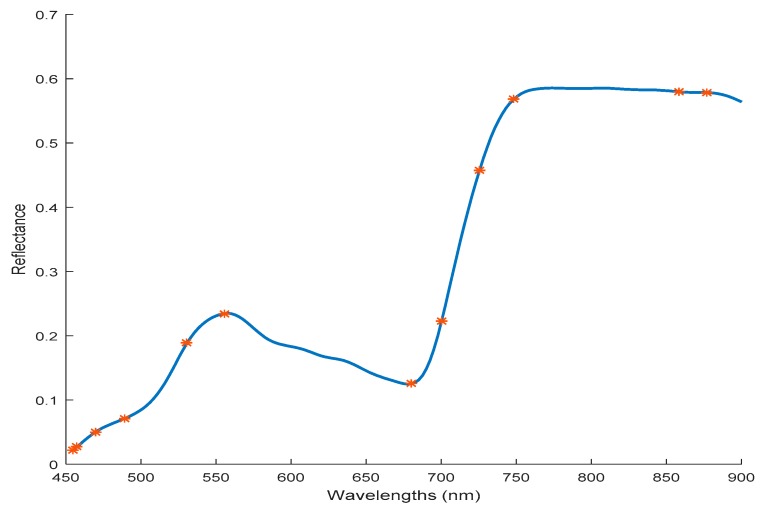
Effective variables selected by successive projections algorithm (SPA).

The characteristic variables selection process by SPA was implemented by comparing the root mean square error of calibration values (RMSEC) values under different variable numbers from 1 to 30. As shown in Figure 6, the RMSEC decreased rapidly when the number of wavelengths increased from 1 to 9, then decreased slowly to the minimum of 0.724, which correspond to the number of effective wavelengths of 12. Since more variables included in the model will decrease model calculation speed, when the RMSEC did not decrease significantly, the number of selected effective wavelengths was determined. The wavelengths selected finally by SPA are shown in Figure 7, which were 450, 462, 481, 523, 548, 690, 715, 740, 758, 850, 868, and 889 nm. The number of selected dielectric variables was 4.68% of the 256 variables in the full spectra.

### 3.4. Comparison and Analysis of the Modeling Results

The BPNN regression models were established, by taking the full spectrum and effective wavelengths extracted by the PCA-loadings method and SPA method as input variables, respectively, and the SPAD value of the wheat leaves as output variables. The modeling results are shown in Table 1. The *RPD* of PCA-loadings -BPNN was 3.363, higher than the 3.259 of SPA-BPNN, and its *RMSEP* was 1.067, which was lower than the 1.101 of SPA- BPNN. When the full spectra were used as inputs, the lowest *RPD* of 1.692 was obtained in BPNN. It can be concluded that the model based on the full spectrum did not show superiority, indicating that the full spectrum not only contained useful information, but also contained noise, which affected the accuracy of the model. Furthermore, the effective bands extracted by SPA and PCA greatly reduced the input variables of the model (about 96% of the variables were deleted) and improved the modeling effect (*R_C_*^2^, *R_P_*^2^, and *RPD*). Among the developed models, the BPNN model combined with the PCA-loadings method was best, as it had the highest *RPD* (3.363), *R_C_*^2^ (0.921), *R_P_*^2^ (0.918) and the lowest *RMSEP* (1.067). Moreover, the number of effective wavelengths extracted by this model accounted for only 3.12% of the total number of wavelengths, simplifying the models and improving the rate of operation greatly. In light of the good modeling effect and minimal number of variables in the PCA-loadings–BPNN method, this method was adopted in the subsequent visualized analysis.

### 3.5. Visualization of Chlorophyll Content Maps

**Figure 8:**
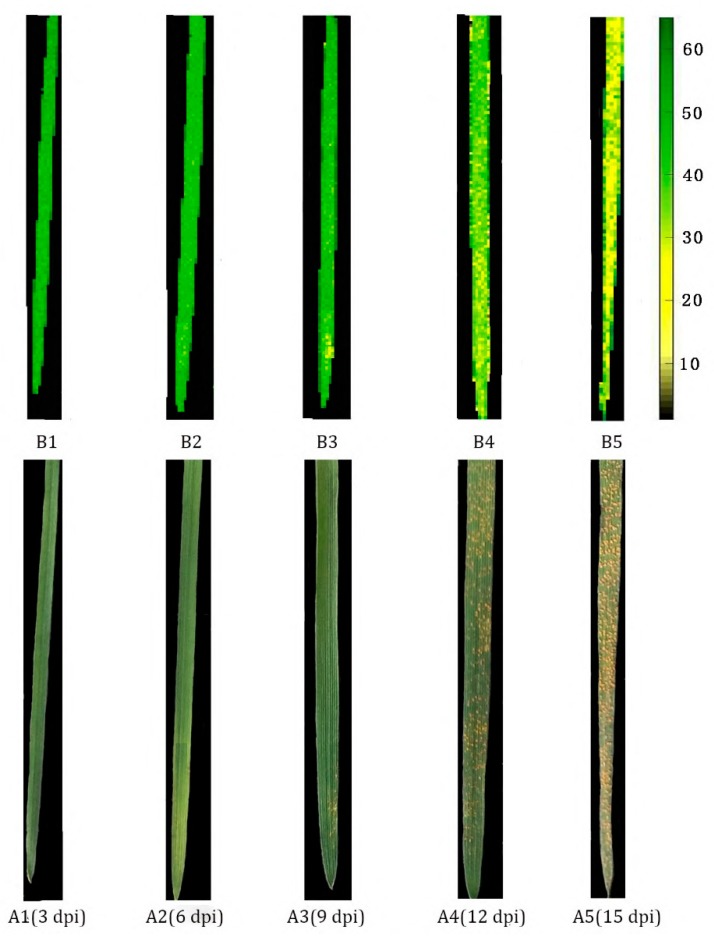
Chlorophyll distribution of wheat leaves during different days past inoculation.

The effective bands of each pixel in the hyperspectral images of wheat leaves at different days post-inoculation were extracted and input into the optimal PCA–loadings–BPNN model to calculate the SPAD value of each pixel of the wheat leaves. Then, the SPAD distribution maps of wheat leaves were drawn through pseudo-color processing technology. It means that pixels having similar spectral features offered the same predicted SPAD value, which was then visualized in a similar color in the image. Figure 8 shows the chlorophyll content distributions in the wheat leaves in response to *Pst* infection. Color bars were generated with different SPAD values from small to large (0–60) shown in a different color from black to green.

RGB images of the wheat leaves with five disease stages are shown in Figure 8 (A1–A5, from left to right, early incubation sample (3 dpi), mid-term incubation sample (6 dpi), late incubation sample (9 dpi), diseased sample (12 dpi), severe diseased sample (15 dpi)). No obvious symptoms appeared in early incubation period (Figure 8, A1) and mid-term incubation period (Figure 8, A2). With the increase of infection day, more and more yellow urediospores appeared on the wheat leaves (Figure 8, A3). The diseased leaves were visually covered by a mass of yellow-colored stripes (Figure 8, A4, A5). Therefore, it is difficult to diagnose the early infection of the fungus according to the RGB images until obvious symptoms appear.

Figure 8 also shows the chlorophyll distribution maps in different stages of wheat leaves with 17 × 148 pixels of each sample, corresponding to RGB images. Different colors in the distribution map represented different values of SPAD in the image in proportion to the spectral differences of the corresponding pixels. As the infection days increased, the colors of the images were gradually shifting from green to yellow, which obviously reflected the decrease of SPAD and the presentation of *Pst* status during the infection process. For example, when the SPAD values of the whole leaf were homogeneous (Figure 8, B1), and there was no abnormal point, the wheat sample could be judged as healthy or at the early stage of infection process. With the increasing days of the *Pst* infection, some yellow spots with low SPAD value appeared, and the colors were fairly nonuniform along with different locations of wheat samples (Figure 8, B2, B3). When a lot of yellow spots appeared in the chlorophyll distribution maps, it revealed that the chlorophyll on the leaves was generally severely damaged, and the chlorophyll content decreased greatly. At this time, the leaves were in the stage of diseased (Figure 8, B4, B5). 

In particular, several little yellow spots could be identified in the visualized distribution diagram on day 6 post-inoculation. However, no obvious disease spots appeared in the RGB image. It could be concluded that the disease spots were detected in the chlorophyll content distribution maps at mid-term incubation period (6 dpi), which appeared three days earlier than the time of symptomatic appearance. The subtle changes are impossible to be observed by the naked eyes in early stage, but can be detected through the chlorophyll maps, thus, it is very useful and meaningful for the better understanding of the dynamic changes of chlorophyll content in wheat leaves during *Pst* infection process and is also helpful and important for the early detection of wheat stripe rust. 

## 4. Discussion

**Figure 9:**
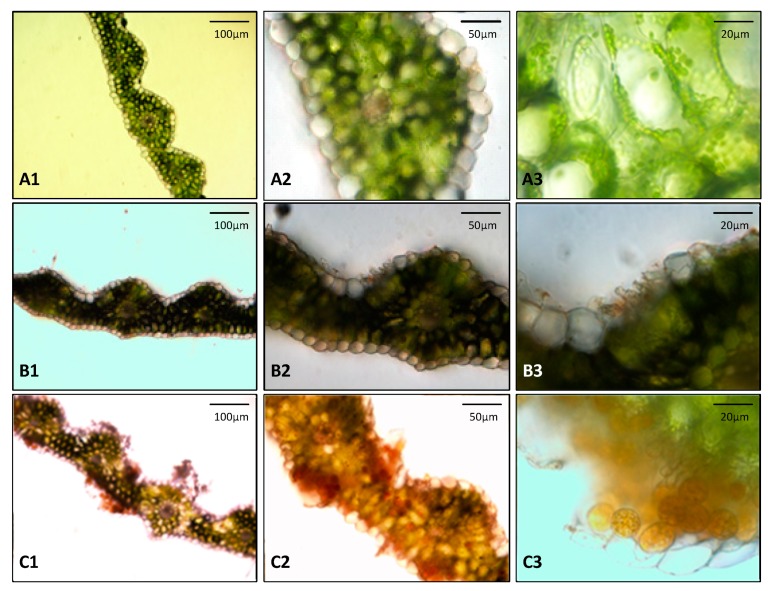
Microscopic images of wheat leaf tissue during different stages of pathogen development. (**A**) healthy tissue; (**B**) tissue at the infection site at 8 dpi without macroscopic symptoms but with the epidermis layer destroyed; and (**C**) chlorotic tissue at 15 dpi with *Pst*.

Disease symptoms commonly reflect the interaction between the plant and pathogen. Once the pathogen successfully infects the leaves, a series of external morphological and internal physiological changes turn up during the process. Figure 9 depicts the microstructure images of the anatomical structure of normal wheat leaves and leaves affected by stripe rust for eight days and fifteen days. It can be seen that the healthy leaves had a complete structure of crosscut sections, a compact structure, and plentiful epidermal cells, and the chloroplasts were attached around the cell wall (Figure 9A). When leaves were infected by the pathogens, the uredospores of stripe rust broke through the protection of the epidermal cells, then entered the tissues and destroyed the internal structure (Figure 9B). The epidermis, dermis, and chloroplast cells of the diseased leaves were severely damaged (Figure 9C). From the perspective of pathology, the leaf structure and cell tissue of wheat leaves infected by stripe rust were damaged, resulting in a decreased photosynthetic efficiency and chlorophyll concentration. Researches have shown that the specifics of these symptoms change the spectral and spatial information of plants. Thereby, it was feasible to use hyperspectral imaging technology for the diagnosis of wheat stripe rust, which provides a new approach for the real-time detection of wheat stripe rust.

When wheat leaves are attacked by *Puccinia striiformis f. sp. tritici*, there will be a long incubation period, without any obvious symptoms. The variation of pigments in infected cells is one of the most important characteristics associated with the disease severity of plants, due to pigments tend to vigorous plant organism. Therefore, the visualization of chlorophyll content distribution is helpful and meaningful to observe the *Pst* disease at an early stage. 

Traditionally, measurement of chlorophyll content based on chemical analysis, such as ultraviolet spectrophotometry and high performance liquid chromatography, involve labor-intensive, time-consuming and tedious extraction procedures. Recent studies have demonstrated the feasibility of retrieval of chlorophyll content from hyperspectral vegetation indices composed by the reflectance of specific bands [52]. But this technique provides spectrum information with low spatial resolution, which fails to provide the spectral details at each pixel on targets images. Since it is quite difficult to measure the chlorophyll content for every part of the sample using chemical analysis methods or vegetation indices, hyperspectral imaging makes it feasible to detect the disease spot in the chlorophyll content distribution maps at early stage of stripe rust, by building visual color images to display the spatial distribution of chlorophyll content in heterogeneity. However, no obvious disease spots appears in the RGB image at the same time, as shown in Figure 8.

Besides the fungal infection, the pigments disorder of leaves could also be caused by insects and nutrient deficiencies. For example, nitrogen deficiency can cause a low chlorophyll density distribution on leaves [36]. In this case, however, the symptom is usually stabilized, and would never spread over the infected time. Additionally, some insects can cause significant damage by eating mesophyll. Nevertheless, the destroyed area is always expressed as a worm eaten path or wormholes that contain no pigment. These characteristics of both stresses are mostly different from that of diseases. 

The pathogenesis of wheat stripe rust and its corresponding control measures and methods have been increasingly mature in China. However, the early monitoring and prediction of this disease is still rare. Since wheat stripe rust has no obvious symptoms in the early stage, and when visible characteristics of the disease shows on wheat leaves, the yellow rust spores are dispersed by the wind and can result in rapid disease development in adjacent wheat crops. Therefore, in the case of unknown susceptibility, how to realize the early monitoring of infection plants have important practical significance and application value for the prevention and control of wheat stripe rust disease. In this study, continuous spectral monitoring of healthy and infected wheat leaves, combined with the synchronous analysis of physiological and biochemical parameters (such as chlorophyll content) in the corresponding period, could accurately identify infected wheat leaves at an early stage of the disease. This provides a basis for further research into the early monitoring and forecasting of wheat stipe rust using hyperspectral imaging technology. 

However, due to the limitations of time and test conditions, the results were obtained under ideal laboratory conditions in this study. Generally speaking, in the field environment, wheat infected with stripe rust has two months or longer incubation period. In the in-situ conditions, the prolongation of latent time, the complexity of the environment and the increase of variables make the research more difficult, but it is the direction and focus of our future research. In future work, more variable factors of in-situ conditions should be considered, such as wheat varieties, field environment, soil, light, water and other factors, to develop more adequate models for early detection of wheat stripe rust.

## 5. Conclusions

This research was conducted to estimate the chlorophyll content distribution in wheat leaves during the incubation period of wheat stripe rust using hyperspectral imaging in the region of 450–900 nm. After acquiring the hyperspectral data, the PCA–loadings and SPA methods were employed to select the effective wavelengths, based on which the BPNN model were established for predicting SPAD value. Among all of the proposed models, the PCA–loadings–BPNN had a simple structure (containing only eight variables) and good performance. The modeling accuracy (*R_C_*^2^) and validation accuracy (*R_P_*^2^) were 0.921 and 0.918, respectively, and the *RPD* was 3.363. This optimal model was used to predict the SPAD values of each pixel for visualizing their distribution in wheat leaves. The SPAD distribution diagram of wheat leaves at different days post-inoculation could intuitively reflect the dynamic response relation of the chlorophyll content and the hyperspectral reflectance in wheat leaves infected with stripe rust, and the visualized detection method identified spores on the sixth day post-inoculation, which was three days earlier than the macroscopic observation, thus providing a new method for the early detection of wheat stripe rust.

## Figures and Tables

**Table 1:** Performance results based on different regression models.

Model	Variables Number	Calibration Sets		Prediction Sets		
		*R_C_* ^2^	*RMSEC*	*R_P_* ^2^	*RMSEP*	*RPD*
FULL–BPNN	256	0.907	1.739	0.898	1.837	2.934
PCA–BPNN	8	0.921	0.986	0.918	1.067	3.363
SPA–BPNN	12	0.924	0.989	0.917	1.101	3.259
